# Predictive Factors of Radioactive Iodine Therapy Refractoriness in Patients with Differentiated Thyroid Carcinoma

**DOI:** 10.1055/s-0044-1787731

**Published:** 2024-06-14

**Authors:** Majdouline Bel Lakhdar, Ayat Mouaden, Mourad Zekri, Dounia Alami, Hamza Zarouf, Imad Ghfir, Hasnae Guerrouj

**Affiliations:** 1 Department of Nuclear Medicine, Academic Hospital Ibn Sina, Faculty of Medicine and Pharmacy, University Mohammed V, Souissi, Rabat, Morocco

**Keywords:** differentiated thyroid carcinoma (DTC), radioactive iodine therapy (RAI), RAI refractoriness (RAIR), predictive factors

## Abstract

**Aim** Differentiated thyroid carcinoma (DTC) is the most prevalent endocrine malignancy, with radioactive iodine (RAI) therapy being a standard of care. However, RAI refractoriness, occurring in a subset of patients, significantly impacts survival rates. Understanding predictive factors for RAI refractoriness is crucial for optimizing patient management.

**Methods** This retrospective study analyzed data from 90 DTC patients at Ibn Sina University Hospital, Morocco. Patients were categorized into RAI-refractory (RAIR) and non-RAIR groups based on established criteria. Statistical analyses, including univariate and multivariate logistic regression, were performed to identify predictive factors of RAI refractoriness.

**Results** Age at the time of diagnosis ≥ 54 years, primary tumor diameter ≥ 29 mm, and distal/nodal metastasis were independent predictors of RAIR-DTC. Additionally, the oncocytic carcinoma histological subtype significantly increased the risk of refractoriness. These findings were consistent with previous studies and underscored the importance of early detection and risk stratification.

**Conclusion** Recognition of predictive factors for RAI refractoriness, including age, tumor size, distal/nodal metastasis, and histological subtype, facilitates early identification of high-risk patients. This enables timely intervention and personalized treatment strategies, particularly relevant in resource-limited settings. Further prospective studies are warranted to validate these findings and explore additional molecular markers for improved prediction of RAI refractoriness.

## Introduction

The most frequent endocrine malignancy is thyroid cancer (TC). Differentiated thyroid carcinoma (DTC) accounts for more than 90% of TC 1 and includes follicular thyroid carcinoma (FTC), invasive encapsulated follicular variant papillary thyroid carcinoma (PTC), PTC, oncocytic carcinoma (OCA) of the thyroid, and follicular-derived carcinomas: high-grade poorly DTC (PDTC) and differentiated high-grade thyroid carcinoma (DHGTC). 2

DTC exhibits a wide range of clinical manifestations, treatment responses, and outcomes, which can be influenced by factors such as gender, age, and ethnicity. Understanding these variations is crucial for personalized treatment strategies and for improved outcomes. Females have a higher incidence of DTC compared with males, yet they often experience better outcomes, which may be related to reproductive hormones. 3 Age also plays a significant role in prognosis, with older age at diagnosis (age ≥ 55 years) associated with poorer outcomes. 4 5 On the other hand, black Americans tend to have worse overall survival rates compared with white Americans, 6 while Arabic patients experience DTC onset approximately 10 years earlier than the global population average. 5

Surgery, thyroid-stimulating hormone (TSH) suppression therapy, and radioactive iodine (RAI) therapy to eliminate any remaining normal thyroid tissue or thyroid micrometastases are the standard treatments for DTC. With an excellent 10-year overall survival rate of more than 90%, this multimodal therapy can produce positive results. 7 8 However, RAI refractoriness develops in 5 to 15% of DTCs and 50% of metastatic cases, lowering the 10-year survival rate to around 20%. 9 10

Refractoriness has been linked to advanced age, large metastases, low degree of differentiation, and high fluorodeoxyglucose uptake. 11 At the molecular level, it has been hypothesized to be related to decreased sodium iodide symporter (NIS) and thyroid peroxidase (TPO) expression. 12 13 Reduced NIS expression impairs TC cells' capacity to concentrate RAI, while decreased TPO expression slows radioiodine's oxidation and shortens its effective half-life. 12 According to numerous reports, genetic or epigenetic changes have a significant role in the initiation, progression, and dedifferentiation of PTC, particularly through the activation of the phosphatidylinositol-3 kinase (PI3K) and mitogen-activated protein kinase (MAPK) signaling pathways. 14 Based on this molecular pathophysiology, novel and promising approaches have been attempted to restore the expression of iodine-metabolizing genes and they become additional options to local treatments that include external beam radiation, radiofrequency ablation, cryoablation, and embolization. 15

Early detection of RAI-refractory (RAIR)-DTC can help patients avoid needless RAI therapy and allow the transition to other, more successful treatments. This study attempts to uncover predictive factors for the development of RAI refractoriness to more accurately identify patients who require more thorough tumor staging and more effective therapy.

## Materials and Methods

The study, conducted between January 2019 and October 2023, involved a retrospective review of medical records from 300 DTC patients. It was based on the analysis of epidemiological, clinical, histopathological, and imaging data obtained from the database of the Department of Nuclear Medicine at Ibn Sina University Hospital in Morocco.

### Patients

Among the 300 cases, 90 DTC patients with confirmed PTC, FTC, and OCA histology, all of whom had undergone at least one course of RAI treatment, were included. The study excluded 178 patients with incomplete follow-up data and 32 patients who had not yet undergone RAI treatment. The distribution of histological subtypes was as follows: PTC (57%), FTC (20%), OCA (16%), DHGTC (5%), and PDTC (3%). All patients included in the study provided oral and signed informed consent.

Patients were split into two groups: the RAIR group and the non-RAIR group. We used the most recent guidelines from the American and European Thyroid and Nuclear Medicine Societies to define patients with RAIR tumors 16 :

1. Patients with distant metastasis or locoregional recurrence that does not take up RAI at the time of initial treatment.
2. Patients with tumors without RAI uptake or its progressive decline in the posttreatment scan several days after RAI therapy.
3. Patients with more than one metastatic lesion and with no uptake in at least one lesion in the posttherapy scan.
4. Patients with structural progression of tumors 12 to 16 months following RAI therapy even after the presence of iodine uptake in the posttherapy scan.
5. Patients with tumors who have cumulatively received 22.2 GBq or more and show no evidence of remission.

### Statistical Analysis

Statistical analysis was performed using IBM SPSS Statistics software for Windows version 23 (IBM Corp, Armonk, New York, United States).

Continuous data were expressed as the mean ± standard deviation. We used the receiver operating characteristic (ROC) curve to determine the optimal cutoff to predict RAIR disease. We compared variables following a normal distribution with a *t* -test and for variables not following a normal distribution with Mann–Whitney *U* test. Categorical data were expressed as number ( *n* ) and were compared utilizing the chi-squared test or Fisher's exact test. A two-tailed *p* -value of < 0.05 was considered statistically significant. Significant variables selected in the univariate analyses were included in multivariate logistic regression to investigate whether risk factors were independently associated with refractoriness. Odds ratios and 95% confidence intervals were calculated to determine the relevance of all potential predictors.

## Results

### Patients' Characteristics

A total of 90 DTC patients (7 males and 83 females; age range: 17–77 years) were involved in this study, 32 were in the RAIR group and 58 were in the non-RAIR group. Among them, 3 cases had OCA subtype, 9 cases had FTC, and 78 cases had PTC with 3 cases of PTC high-risk subtypes (1 case of tall cell subtype and 2 cases of solid/trabecular subtype). The clinical and pathological characteristics of the included patients are shown in Table 1 . Unless specified otherwise, the term “metastasis” encompasses both distant metastasis and lymph node involvement.

**Table 1:** Univariate analysis of the factors in the RAIR group and non-RAIR group

Baseline factors	RAIR	Non-RAIR	*p*
**Gender** , female/male	30/2	53/5	1.000
**Age (mean ± SD, y)**	53.06 ± 14.128	44.43 ± 13.088	0.005 a
**≥** **54/<** **54**	19/13	16/ 42	0.004 a
**Metastasis** , yes/no	18/14	4/54	0.000 a
**Tumor diameter (mean ± SD, mm)**	40.688 ± 22.4979	19.914 ± 13.1913	0.000 a
**≥** **29/<** **29**	21/11	14/44	0.000 a
**ETE** , yes/no	3/29	4/54	0.696
**Multifocality** , yes/no	12/20	14/44	0.226
**Histology PTC**	25	53	0.106
**High-risk PTC subtype** , yes/no	2/23	1/52	0.239
**FTC**	4	5	0.716
**OCA**	3	0	0.042 a

Abbreviations: ETE, extrathyroid extension; FTC, follicular thyroid cancer; OCA, oncocytic carcinoma; PTC, papillary thyroid carcinoma; RAIR, radioiodine-refractory differentiated thyroid carcinoma; SD, standard deviation.

a Statistically significant difference.

### Univariate Analysis

Age, initial tumor size, metastasis, and OCA histological subtype all demonstrated statistically significant differences between the two groups in the univariate analysis. Gender, extrathyroidal extension, primary tumor multifocality, FTC, PTC, and its high-risk subtypes did not show any statistically significant differences ( Table 1 ).

### ROC Curve Analysis

**Fig. 1:**
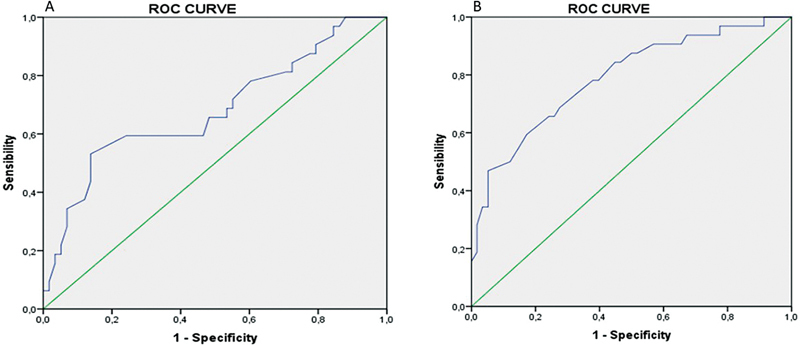
Receiver operating characteristic (ROC) curve to predict the prevalence of radioiodine refractory cancer. ( **A** ) ROC curve for age, area under the curve (AUC) = 0.681, cutoff = 54 years with sensibility of 59.4% and specificity of 75.9%. ( **B** ) ROC curve for tumor size, AUC = 0.790, cutoff = 29 mm with sensibility of 65.6% and specificity of 75.9%.

ROC curve was used to determine the optimal cutoff value to predict RAIR-DTC in terms of age at diagnosis and primary tumor diameter, which were 54 years old (area under the curve [AUC] = 0.681) and 29 mm with (AUC = 0.790), respectively ( Fig. 1 ).

### Multivariate Logistic Regression Analysis

Multivariate logistic regression ( Table 2 ) revealed that age, primary tumor diameter, and metastasis were independent factors in predicting RAIR-DTC, specifically age over 54 years old, primary tumor diameter over 29 mm, and the presence of metastasis.

**Table 2:** Multivariable logistic regression

	Odds ratio	95% CI	*p-* Value
*Age ≥* *54/<* *54* *y*	6.502	1.832–23.080	0.004 a
*Tumor diameter ≥* *29/<* *29 mm*	6.666	1.962–22.641	0.002 a
*Metastasis* yes/no	22.553	5.099–99.752	0.000 a

Abbreviation: CI, confidence interval.

a Statistically significant difference.

Due to the absence of OCA histological subtype cases in the non-RAIR group, this variable was not included in the logistic regression analysis.

## Discussion

RAI ablation following total thyroidectomy has long been considered the gold standard of therapy for the majority of DTC cases in several nations, including Morocco. The current RAIR-DTC diagnosis is based on post-RAI therapy evaluation, which raises the likelihood that patients underwent unneeded RAI and thus missed the opportunity to benefit from more efficient treatments such as targeted medication therapy. Therefore, it is crucial to be able to anticipate RAI refractoriness before RAI therapy. 17 Our study is the first to look into the risk factors for RAIR-DTC in Moroccan patients.

Age at diagnosis, initial tumor diameter, distal/nodal metastasis, and OCA histological subtype were all found to be significant predictors of RAIR-DTC in our study. Multivariate logistic regression analysis supported three independent predictors of RAIR-DTC: age at diagnosis ≥ 54 years, initial tumor diameter ≥ 29 mm, and distal/nodal metastasis. OCA subtype was not included in the logistic regression due to insufficient cases in the non-RAIR group.

Age is a recognized independent prognostic factor for RAI therapy effectiveness. 18 19 20 Our study showed that age (cutoff 54 years old) is an independent predictor of refractoriness and this was consistent with two Chinese studies conducted by Chai et al 1 (cutoff value was 40 years old in the thyroglobulin antibodies [TgAb] negative group) and Liu et al 21 (cutoff was 48 years). Another Chinese study by Li et al 22 of 224 patients in the non-RAIR group and 112 patients in the RAIR group discovered that refractoriness risk was significantly increased with age (cutoff was 55 years), but this finding was not supported by a logistic regression model and was not taken into account in the scoring system for predicting RAIR-DTC. According to a German study by Kersting et al, 23 age > 55 years was a risk factor for RAI refractoriness in PDTC. A Japanese study of 258 patients by Nakanishi et al 24 revealed that the prevalence of RAI uptake was 41.5% for patients younger than 55 years, but this decreased significantly to 8.1% for those 55 years or older. All of these results could be attributed to the weakened immune system, decreased radiation sensitivity, decreased expression of NIS, and inadequate uptake of ^131^ I that older patients experience. 1 25 Children's thyroid tissue has smaller follicles and increased expression of NIS, pendrin, and dual oxidases, according to Faggiano et al's findings. 26 Additionally, Mihailovic et al 27 discovered that younger patients had a higher likelihood of having RAI-avid distant metastases. Therefore, to prevent unneeded or excessive RAI therapy, thorough follow-up monitoring and/or additional management methods should be implemented for elderly patients. 1

In DTC, the primary tumor diameter has been identified as a predictor of prognosis. 28 According to our study, primary tumor diameter (≥ 29 mm) was an independent predictive factor of RAI refractoriness. Other studies support our findings. Li et al 22 found that primary tumor size > 10 mm and primary tumor size > 20 mm significantly increased the risk of RAIR cancer. Kersting et al 23 showed that primary tumor size > 40 mm was significantly associated with late occurrence of RAIR disease. Liu et al 21 conducted a retrospective study on 404 patients and demonstrated that primary tumor diameter > 18.5 mm significantly increases the risk of RAIR-DTC. Additionally, Liu et al 29 reported that patients with advanced tumor stage had a higher RAIR rate. In a study conducted by Lee et al, 30 which was in line with a previous report by Tavares et al, 31 NIS expression was inversely correlated with tumor size, this finding implies that NIS depletion occurs as a tumor progresses and that NIS downregulation is likely mediated by molecular mechanisms occurring in the late stages. 30

Histology has been known as a significant prognostic indicator in PTC. 32 According to a meta-analysis conducted by Luo et al, 32 the presence of high-risk histological subtypes such as tall cell variation PTC, sclerosing diffuse PTC, hobnail variant PTC, FTC, OCA, and PDTC is a predictor of RAIR-DTC. Some studies illustrated that FTC, OCA, and PDTC were more likely to become resistant to RAI treatment. 33 34 35 36 In our study, we found that OCA histological subtype significantly increased the risk of RAI refractoriness, which is consistent with the literature where it is believed that RAI therapy is unlikely to be helpful in this histological subtype since it does not accumulate RAI. 37 38 39 40 Despite the rarity of studies shedding light on the molecular basis of OCA, these results may be explained by the inhibition of NIS expression, which is most likely connected to an activation of the PI3K-Akt-mTOR pathway. 41 42

DTCs with distant metastases have less favorable outcomes, 43 and the effectiveness of I-131 therapy is adversely correlated with the size of metastatic lesions. 44 According to the results of our investigation, metastasis at any site, including the lymph nodes, lungs, or bones, is an independent predictor of RAI refractoriness. In the study conducted by Li et al, 22 lymph node metastasis number (≥ 4), lymph node metastasis rate (≥ 53%), and pN stage (N1) were included in the scoring system for predicting the prevalence of RAIR cancer. Liu et al 21 demonstrated that the site of metastasis displayed highly independent associations with RAIR-DTC. According to Schlumberger et al, 45 only 42% of patients with metastases demonstrate significant RAI uptake, even after appropriate stimulation by TSH and in the absence of excess iodine. The underlying mechanisms of these findings are not well understood, but hypoxia in large tumors may contribute to RAI resistance. 44 The radiation dose delivered to extrathyroidal tissues is typically 1,000 to 10,000 times lower than that delivered to the thyroid gland and is another factor limiting RAI efficacy in metastatic sites. 46

Parallel studies focused on the molecular, genetic, and epigenetic basis to predict RAI refractoriness. The underlying mechanisms behind RAI resistance are mostly represented by decreased expression of NIS, diminished membrane targeting of NIS, or both, which are mainly caused by genetic and epigenetic aberrations in the RTK/BRAF/MEK/ERK and PI3K-AKT-mTOR pathways. 47

The BRAFV600E mutation is one of the most well-known mutations that abnormally activate the MAPK pathway. BRAF activation has been shown to inhibit NIS expression in two ways: The first is the stimulation of transforming growth factor (TGF)-β-Smad3 signaling, which limits the ability of thyroid-specific transcription factor PAX8 (paired box gene 8) to bind to the NIS promoter in follicular cells. The second way involves histone deacetylation of the NIS promoter's H3 and H4 lysine residues, which prevents transcription. 32 47 Azouzi et al 48 discovered that a BRAF mutation regulates nicotinamide adenine dinucleotide phosphate oxidase 4 (NOX4) expression via the TGF-β/Smad signaling pathway, and that NOX4-dependent reactive oxygen species generation plays an important role in the lowering of NIS expression in BRAF-mutated PTC.

A study conducted by Chai et al 1 to predict RAI resistance, concluded that the mutated BRAF gene was an independent predictor for RAIR-DTC in the TgAb-negative group and was an influencing factor in the TgAb-positive group.

García and Santisteban reported that insulin-like growth factor (IGF)-1 could inhibit TSH/forskolin-induced NIS expression through activation of the PI3K/AKT signaling pathway in thyroid cells. 49

The expression of the tumor-promoting growth factor (IGF-2) was noticeably increased in RAI-resistant tumors according to Crezee et al, 50 who proposed that IGF-2 may be a contributing factor in the emergence of RAI refractoriness.

Another study conducted by Liu et al 29 included ribonucleic acid biomarkers (IPCEF1 and hsa-mir-486–5p) in a prognosis prediction model to predict the progression-free survival of PTC patients with RAI therapy.

Our study has certain drawbacks. First, because this study was a retrospective assessment, there may have been a bias in the selection of the control group. Second, because of the small sample size and the fact that all patients came from a single center, we were unable to generalize the findings. Therefore, a confirmation by future prospective studies and larger collectives in multiple centers is needed to allow generalization. This study is also limited by the fact that its scope is narrow, centered on just a few clinicopathologic factors while disregarding numerous other potential confounders such as biochemical markers and imaging patterns. Also, due to the limited resources, we were unable to incorporate molecular and genetic studies; however, we are considering doing so in the future.

## Conclusion

Our study revealed that age at diagnosis, initial tumor diameter, distal/nodal metastasis, and OCA histological subtype were significant predictors of RAI refractoriness. Among these factors, only three were found to be independent predictors: age at diagnosis ≥ 54 years, initial tumor diameter ≥ 29 mm, and distal/nodal metastasis. These findings emphasize the importance of early identification of high-risk patients to enable timely intervention and personalized treatment strategies. Future prospective studies with larger sample sizes and incorporating molecular and genetic analyses are warranted to validate these findings and explore additional predictive markers for improved management of RAIR DTC.
